# Reviews of Environmental Health, 2004

**Published:** 2004-06

**Authors:** James Burkhart

**Affiliations:** National Institute of Environmental Health Sciences, National Institutes of Health, Department of Health and Human Services, Research Triangle Park, North Carolina, USA

Our broadening realization of the interconnectivity of well-being among species and ecosystems must bring new perspective to environmental health if we are to deal successfully with the dynamics of global change and human activity. Individuals might have interest and expertise in particular areas, but understanding the actions we must pro-actively take to enhance human and environmental health requires us to integrate many fields into articulated direction. *EHP* is committed to facilitating interdisciplinary communication from the most basic biological mechanism to public policy issues in the interest of health.

Realistically an individual is challenged by the prospect of seeing the broad patterns that might come from integrating interdisciplinary information. Language, hypotheses, methods, and data interpretation vary greatly across fields. The rapid evolution of new technologies in all disciplines produces even more daunting challenges to environmental scientists, educators, and policymakers. Yet this integration is profoundly important to the individual disciplines because larger views can produce more relevant questions and direction within each field of study.

The scientific complexity of environmental health in this time can produce confusion, or as we would hope, excitement and enthusiasm about discovery and our capacity to affect change in a positive direction. There are many important decisions and debates that must be met in the near future by the environmental health community. These involve global and local environments, exposure of humans and wildlife to new and old pollutants, children’s health, environmental medicine, conservation biology, and emerging technologies. The objective of the *EHP* Annual Review issue is to provide the latest information on particular topics in a way that is useful both to a very broad readership and to the specialists. As the Annual Review issue continues to evolve, *EHP* would like to engage the environmental health communities to consider, suggest, and submit for consideration reviews that summarize new developments in environmentally relevant areas and provide balanced background and perspective.

The review by Theo Colborn (2004) in this issue explores the role of environmental contaminants in disrupting thyroid signaling and its impact on the developing brain. The health issues associated with thyroid-mediated developmental disruption transcend humans and wildlife. Developmental pathways are conserved across diverse phylogenies. It is possible that classes of environmental contaminants capable of altering development and behavior in species such as amphibians may also contribute to attention deficit hyperactivity disorder and behavioral problems for humans in developed countries.

Kamel and Hoppin (2004) continue to explore the role of pesticide exposure and neurotoxicity. Read about the unresolved issues surrounding acute versus chronic exposure and the potential for changes in neurobehavioral performance reflecting cognitive and psychomotor dysfunction. The issues are pivotal if we are to plot a course toward better environmental health quality.

The increasing human population produces enormous waste disposal problems and responsibilities. All those interested should find the review by Rideout et al. (2004) of value, as it explores the possibility of accumulating human exposure to polychlorinated dibenzo-*p*-dioxins and polychlorinated dibenzofurans through sewage sludge recycling as a nutrient source in agriculture.

Learn from the review by Armstrong et al. (2004) the issues associated with occupational exposure to polycylcic aromatic hydrocarbons and lung cancer, together with how the results might influence risk assessment.

A large percentage of the world human population lives close to coastlines, and human activity has placed many of those ecosystems at serious risk. The ecologic stress has increased toxic substances and pathogens. Niemi et al. (2004) provide the recent developments and suggest we need to have new coastal ecologic indicators if we are to successfully protect environmental health.

We all are aware of the potentially devastating effects of lead exposure on children and the recent successes in reducing lead in the environment. Now learn from Koller et al. (2004) about the possibilities for intellectual impairment at low levels and the relationships of other environmental factors in the health outcome.

Mini-monographs containing up to six manuscripts dealing with specific topics within a broadly important topic have increased in popularity over the last year in *EHP*. We seek to weave together different aspects of a larger topic so that the readers can come away with perspectives not easily attainable with a single review. Here we have included the mini-monograph “Health and Environment Information Systems for Exposure and Disease Mapping, and Risk Assessment” (EHP 2004) because it will give readers the opportunity to understand contributions of new geographic information systems (GIS) technology to environmental health and exposure tracking. The importance of this technology lies in the fact that exposures to large numbers of potentially toxic agents are uneven geographically and temporally. The GIS might be used to provide more rapid exposure and risk assessments in order to better protect humans and the environment.

The reviews and monograph articles included here should be of interest to the broad environmental health community. Your comments, insights, and suggestions for future directions would be appreciated.
